# Multi-Step Mechanical and Thermal Homogenization for the Warpage Estimation of Silicon Wafers

**DOI:** 10.3390/mi15030408

**Published:** 2024-03-18

**Authors:** Zhouyi Xiang, Min Chen, Yonghui Deng, Songhua Huang, Sanli Liu, Ji Li

**Affiliations:** 1 School of Advanced Technology, Xi’an Jiaotong-Liverpool University, Suzhou 215123, China; zhouyi.xiang22@student.xjtlu.edu.cn (Z.X.); songhua.huang@xjtlu.edu.cn (S.H.); sanli.liu22@student.xjtlu.edu.cn (S.L.); 2 ZINSIGHT Technology (Shanghai) Co., Ltd., Shanghai 201114, China; yonghui.deng@zinsight-tech.com; 3 Key Laboratory of MEMS of the Ministry of Education, Southeast University, Nanjing 210096, China; j.li5@seu.edu.cn

**Keywords:** wafer warpage, integrated capacitor, multi-step mechanical homogenization, multi-step thermal homogenization

## Abstract

In response to the increasing demand for high-performance capacitors, with a simultaneous emphasis on minimizing their physical size, a common practice involves etching deep vias and coating them with functional layers to enhance operational efficiency. However, these deep vias often cause warpages during the processing stage. This study focuses on the numerical modeling of wafer warpage that occurs during the deposition of three thin layers onto these vias. A multi-step mechanical and thermal homogenization approach is proposed to estimate the warpage of the silicon wafer. The efficiency and accuracy of this numerical homogenization strategy are validated by comparing detailed and homogenized models. The multi-step homogenization method yields more accurate results compared to the conventional direct homogenization method. Theoretical analysis is also conducted to predict the shape of the wafer warpage, and this study further explores the impact of via depth and substrate thickness.

## 1. Introduction

Wafers integrated with capacitors play a crucial role in the manufacturing of Micro Electro Mechanical Systems (MEMS). Silicon-based discrete capacitors are currently under investigation as a potential method to improve overall operational efficiency by providing better equivalent series inductance (ESL) performance compared to conventional ceramic capacitors [1,2,3]. The Through Silicon Via (TSV) capacitor, commonly utilized in Si wafers, is created by etching deep vias into the silicon substrate. This enables the attainment of a significantly higher capacitance density and the formation of compact structures [4,5].

Nevertheless, the production of high-quality wafers is accompanied by various challenges [6]. Among these challenges, one significant issue is the mitigation of wafer warpage to enhance the efficiency of subsequent processes. Wafer warpage is identified as a primary factor leading to process and device failures, including delamination, cracking, and a decline in device performance [7,8]. Various factors influence the warpage of wafers, such as the mismatch in the coefficient of thermal expansion (CTE) among different materials, fluctuations in film thickness, and variances in pattern density [9,10]. Hence, it is essential to optimize the process parameters to minimize wafer warpage.

An experiment is the most direct method for determining the warpage value. However, conducting numerous physical experiments to quantify wafer warpage is a time-consuming and economically inefficient process. Consequently, there is an urgent and practical need to employ the finite element analysis (FEA) method for simulating wafer warpage. Simulating a wafer with millions of vias would overwhelm current computational systems due to the substantial computational requirements. On the other hand, modeling only a small portion of the wafer with a few vias proved inadequate, as it resulted in numerical inaccuracies due to the limited displacement of each via and difficulties in establishing suitable boundary conditions for the outer surfaces of the simulated structures. Fortunately, a viable solution was found by adopting a multi-scale approach. This approach involves dividing the simulation into two scales. The homogenized mechanical properties of the via layer can be determined at the meso-scale. Then, at the macro-scale, the overall behavior of the wafer can be simulated. Through this method, researchers can achieve a balance between accuracy and computational efficiency, making it feasible to study the behavior of a wafer with millions of vias without overwhelming the computational resources. 

Che et al. [11] developed a wafer-level FEA modeling approach to simulate the warpage of wafers following annealing. However, the model was limited to only two materials: silicon and copper. Wright et al. [12] employed a multi-scale method to simulate the wafer warpage. In their meso-scale simulation, the remote boundary conditions with “coupled” behavior and sliding-wall boundary conditions were applied to the Representative Volume Element (RVE). The RVE, serving as the smallest micro-scale structure suitable for homogenization, enables the examination of large-scale structures while minimizing computational expenses [13]. This type of boundary condition was unsuitable for representing orthotropic materials and estimating the shear modulus, as it overly constrained the RVE, leading to an exaggerated assessment of elastic properties [14]. Consequently, employing node-to-node periodic conditions becomes essential, allowing distorted deformation of boundary surfaces [15]. Feng et al. [16] used the RVE method to create an equivalent model for the DRAM layer in the simulation. The RVE method allows for the representation of the complicated DRAM layer with a simplified model, making it easier to solve. They found that reducing the dicing pitch resulted in a significant reduction in warpage. The study also analyzed the thermal stress distribution in the bonded wafer and identified the stress release caused by interrupting the wafer continuity as the main factor in reducing warpage. Bacciocchi et al. [17] adopted a multi-step homogenization procedure to predict the mechanical property of the multi-phase porous earth material, and the accuracy was validated by a comprehensive experimental campaign. However, applying multi-step homogenization in predicting wafer warpage is rarely seen.

In summary, utilizing a multi-step homogenization procedure in the context of via-type silicon capacitors is relatively uncommon. We have employed a multi-scale approach, coupled with a multi-step RVE homogenization strategy, to simulate the warpage of silicon capacitors and to conduct a theoretical analysis from the perspective of thin film mechanics. Both numerical and experimental data validate the effectiveness of this novel homogenization method. Furthermore, it has been observed that increasing the via depth results in a more significant wafer warpage. Conversely, a thicker substrate can alleviate wafer warpage, although it leads to a thicker wafer. Moreover, parameter sensitivity analyses demonstrate that while both factors affect wafer warpage, the depth of the vias exerts a more substantial influence on wafer warpage. Adopting this approach equips us with a reliable means to predict wafer warpage, promoting MEMS development.

## 2. Materials and Methods

### 2.1. Manufacturing Process and Parametrized Samples

**Figure 1:**
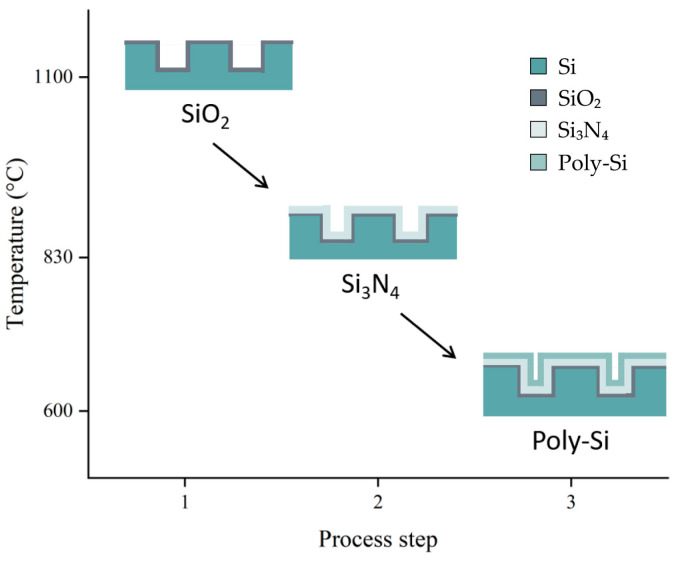
Simplified schematic of manufacturing process. Si substrate; SiO_2_ layer deposition at 1100 °C; Si_3_N_4_ layer deposition at 830 °C; Poly-Si layer deposition at 600 °C.

The sample tested was manufactured using silicon (Si) wafers 150 mm in diameter with a thickness of 725 μm. After an initial cleaning, a hexagonal grid composed of circular openings with diameters of 6 μm and a distance of 3 μm between the nearest neighbors was patterned with lithography. The vias were etched to a depth of 30 μm. Then, a silicon dioxide (SiO_2_) dielectric layer 0.3 μm in thickness was formed by dry thermal oxidation at a temperature of 1100 °C. Next, a 1.6 μm-thick layer of silicon nitride (Si_3_N_4_) was deposited by LPCVD (low pressure chemical vapor deposition) at a temperature of 830 °C. The top electrode was formed by the deposition of in situ n+-doped polysilicon (poly-Si) using LPCVD with a 0.5 μm thickness at 600 °C. The simplified schematic of the manufacturing process is illustrated in Figure 1. It is worth noting that these layers are deposited at varying temperatures, leading to misfit strain at room temperature due to their different CTE, as referenced in [18,19,20,21]. 

**Figure 2:**
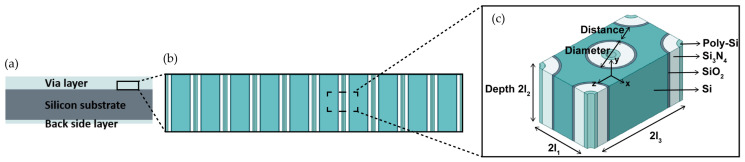
The extraction process and the dimensions of the RVE. (**a**) Side view of the wafer; (**b**) Side view of the via layer; (**c**) Representation of the RVE structure.

The silicon substrate used in this paper has a diameter of 150 mm and a thickness of 725 μm. As seen in Figure 2c, the vias have a diameter of 6 μm, a depth of 30 μm, and a distance of 3 μm between them. The layer thicknesses of SiO_2_, Si_3_N_4_, and poly-Si are 0.3 μm, 1.6 μm, and 0.5 μm, respectively. 

In our simulations, we utilized mechanical and thermal properties as listed in Table 1. These properties have been sourced from existing literature.

### 2.2. Multi-Scale Analysis for Thermal and Mechanical Properties 

Due to the large number of small vias on the wafer, simulating the full geometry model directly is computationally costly. Therefore, it is imperative to adopt a multi-scale method. The initial stage of the multi-scale method involves extracting an RVE from the via layer. The extraction process and the dimensions of the RVE are illustrated in Figure 2. Overall, the multi-scale method comprises two scales of simulations. In the meso-scale simulation, the effective properties of the RVE are determined by using a homogenization method. In the macro-scale simulation, wafer warpage results are calculated by applying the effective properties of the RVE to the via layer. During the deposition process, the dielectric layers will stack on the back side of the substrate simultaneously. These backside layers are modeled as surface coatings in the simulation. However, due to their small thickness (<3 μm) compared to the substrate (725 μm), their impact is minimal.

#### 2.2.1. RVE Homogenization Analysis at the Meso-Scale

The RVE refers to the smallest volume of a material that can be considered representative of the entire material’s behavior [32]. Since the RVE is a part of a periodic material, it is essential to implement Periodic Boundary Conditions (PBC) to ensure that the RVE’s surfaces remain periodic after deformation. Heterogeneity is present at lower length scales of a material. RVE homogenization aims to homogenize the heterogeneity at a lower length scale so that the material can be treated as homogeneous for engineering applications at the upper length scale [15]. This technique is applicable to a wide range of materials, such as composites, lattice structures, and any other material that exhibits spaced periodic repetition. The homogenization method enables the derivation of the effective properties of the complicated via layer from the RVE. Specifically, the objective of homogenization here is to derive the homogenized stiffness matrix and the CTE of the RVE. 

To determine the homogenized stiffness matrix [C] that relates average stress $\left\{\bar{\sigma }\right\}$ and average strain $\left\{\bar{\varepsilon }\right\}$, six static simulations are performed on the RVE with PBC. Equation (1) expresses the correlation between the average stress $\left\{\bar{\sigma }\right\}$ and average strain $\left\{\bar{\varepsilon }\right\}$ using the homogenized stiffness matrix [C].

(1) $$\bar{\sigma_{ij}}=C_{ijkl}\bar{\varepsilon_{ij}}$$

The hypothesis of constant strain energy is employed on the RVE to establish the homogenized stiffness matrix. This ensures that the original and homogenized cells possess equivalent strain energy during deformation. The average strain $\bar{\varepsilon_{ij}}$ is calculated by taking the average of the six applied strain components $\varepsilon_{ij}$ over the volume of the RVE, as described in Equation (2). The homogenized stiffness matrix coefficients can be obtained by solving the six linear elastic equations in Equation (1), as shown in Equation (3).

(2) $$\bar{\varepsilon_{ij}}=\frac{1}{V}\int_{V}\varepsilon_{ij}dV=\varepsilon_{ij}^{0}$$

(3) $$C_{\alpha \beta }=\bar{\sigma_{\alpha }}=\int_{V}\sigma_{\alpha }\left(x_{1},x_{2},x_{3}\right)dV\;\mathrm{where}\;\varepsilon_{ij}^{0}=1$$

Equation (4) presents the constraints for node pairs on opposite faces, where i denotes the direction in the Cartesian system and $u_{i}\left(x,y,z\right)$ represents the displacement of the point $\left(x,y,z\right)$ in the i direction:

(4) $$\left\{\begin{array}{l} u_{i}\left(l_{1},x_{2},x_{3}\right)-u_{i}\left(-l_{1},x_{2},x_{3}\right)=2l_{1}\varepsilon_{i1}^{0}\\ u_{i}\left(x_{1},l_{2},x_{3}\right)-u_{i}\left(x_{1},-l_{2},x_{3}\right)=2l_{2}\varepsilon_{i2}^{0}\\ u_{i}\left(x_{1},x_{2},l_{3}\right)-u_{i}\left(x_{1},x_{2},-l_{3}\right)=2l_{3}\varepsilon_{i3}^{0} \end{array}\right.\;,\;i=1,\;2,\;3$$

Each edge, simultaneously shared by two faces, requires distinct conditions. Equation (5) describes these constraints for edges:

(5) $$\begin{array}{rr} & \left\{\begin{array}{c} u_{i}\left(l_{1},l_{2},x_{3}\right)-u_{i}\left(-l_{1},-l_{2},x_{3}\right)=2l_{1}\varepsilon_{i1}^{0}+2l_{2}\varepsilon_{i2}^{0}\\ u_{i}\left(l_{1},-l_{2},x_{3}\right)-u_{i}\left(-l_{1},l_{2},x_{3}\right)=2l_{1}\varepsilon_{i1}^{0}-2l_{2}\varepsilon_{i2}^{0} \end{array},\;i=1,\;2,\;3\right.\\ & \left\{\begin{array}{c} u_{i}\left(l_{1},x_{2},l_{3}\right)-u_{i}\left(-l_{1},x_{2},-l_{3}\right)=2l_{1}\varepsilon_{i1}^{0}+2l_{3}\varepsilon_{i3}^{0}\\ u_{i}\left(l_{1},x_{2},-l_{3}\right)-u_{i}\left(-l_{1},x_{2},l_{3}\right)=2l_{1}\varepsilon_{i1}^{0}-2l_{3}\varepsilon_{i3}^{0} \end{array},\;i=1,\;2,\;3\right.\\ & \left\{\begin{array}{c} u_{i}\left(x_{1},l_{2},l_{3}\right)-u_{i}\left(x_{1},-l_{2},-l_{3}\right)=2l_{2}\varepsilon_{i2}^{0}+2l_{3}\varepsilon_{i3}^{0}\\ u_{i}\left(x_{1},l_{2},-l_{3}\right)-u_{i}\left(x_{1},-l_{2},l_{3}\right)=2l_{2}\varepsilon_{i2}^{0}-2l_{3}\varepsilon_{i3}^{0} \end{array},\;i=1,\;2,\;3\right. \end{array}$$

Each corner is shared by three faces, leading to their specific constraints given in Equation (6):

(6) $$\left\{\begin{array}{l} u_{i}\left(l_{1},l_{2},l_{3}\right)-u_{i}\left(-l_{1},-l_{2},-l_{3}\right)=2l_{1}\varepsilon_{i1}^{0}+2l_{2}\varepsilon_{i2}^{0}+2l_{3}\varepsilon_{i3}^{0}\\ u_{i}\left(l_{1},l_{2},-l_{3}\right)-u_{i}\left(-l_{1},-l_{2},l_{3}\right)=2l_{1}\varepsilon_{i1}^{0}+2l_{2}\varepsilon_{i2}^{0}-2l_{3}\varepsilon_{i3}^{0}\\ u_{i}\left(-l_{1},l_{2},l_{3}\right)-u_{i}\left(l_{1},-l_{2},-l_{3}\right)=-2l_{1}\varepsilon_{i1}^{0}+2l_{2}\varepsilon_{i2}^{0}+2l_{3}\varepsilon_{i3}^{0}\\ u_{i}\left(l_{1},-l_{2},l_{3}\right)-u_{i}\left(-l_{1},l_{2},-l_{3}\right)=2l_{1}\varepsilon_{i1}^{0}-2l_{2}\varepsilon_{i2}^{0}+2l_{3}\varepsilon_{i3}^{0} \end{array},\;i=1,\;2,\;3\right.$$

The homogenized stiffness tensor is established based on these equations. From the simulation results, the components of the average field $\bar{\sigma_{\alpha }}$ are obtained, and using Equation (3), the coefficients of the homogenized stiffness matrix are derived. Then, the compliance matrix can be obtained by the inverse of the homogenized stiffness matrix.

(7) $$[S]=[C{]}^{-1}$$

Owing to the orthotropic property of the RVE, the compliance matrix is in the following form:

(8) $$[S]=\left[\begin{array}{cccccc} 1/E_{1} & -v_{21}/E_{2} & -v_{31}/E_{3} & 0 & 0 & 0\\ -v_{12}/E_{1} & 1/E_{2} & -v_{32}/E_{3} & 0 & 0 & 0\\ -v_{13}/E_{1} & -v_{23}/E_{2} & 1/E_{3} & 0 & 0 & 0\\ 0 & 0 & 0 & 1/G_{12} & 0 & 0\\ 0 & 0 & 0 & 0 & 1/G_{13} & 0\\ 0 & 0 & 0 & 0 & 0 & 1/G_{23} \end{array}\right]$$

Combing Equations (7) and (8), the equivalent mechanical properties can be acquired as follows:

(9) $$\begin{array}{c} E_{1}=\frac{1}{S_{11}},\;E_{2}=\frac{1}{S_{22}},E_{3}=\frac{1}{S_{33}}\\ G_{23}=\frac{1}{S_{44}},G_{13}=\frac{1}{S_{55}},G_{12}=\frac{1}{S_{66}}\\ v_{12}=-S_{12}\cdot E_{1},v_{23}=-S_{23}\cdot E_{2},v_{13}=-S_{13}\cdot E_{1} \end{array}$$

where E stands for Young’s modulus, v for Poisson’s ratio, G for shear modulus, and S for the coefficient in the compliance matrix. Similarly, the effective CTE can be calculated. By applying a temperature load ∆T to the RVE, the displacements of the RVE in three directions $U_{x},\;U_{y},\;U_{z}$, owing to the thermal expansion, are obtained. Corresponding thermal strains are calculated by the following equation:

(10) $$\varepsilon_{i}=U_{i}/l_{i},\;i=x,y,z$$

Naturally, an effective CTE of the RVE can be acquired as follows:

(11) $${CTE}_{i}=\varepsilon_{i}/∆T,\;i=x,y,z$$

#### 2.2.2. Boundary Conditions for RVE with Void Phase

**Figure 3:**
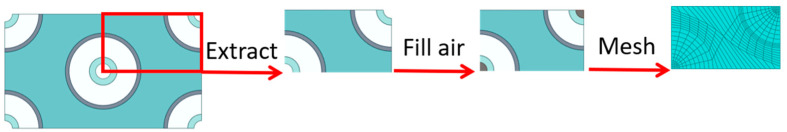
A top view illustration showcasing the RVE model and its homogenization process.

In order to facilitate the creation of constraints mentioned in Section 2.2.1 in Abaqus, the RVE is initially divided into four segments. From these segments, a 1/4 RVE model is then extracted and meshed. The void space within the RVE is filled with elastic air, which has a zero CTE and an elastic modulus that can be ignored [33]. Subsequently, the “radial pattern” command is employed to assemble the complete RVE model, and then the homogenization method can be applied. This process allows for easier identification of node pairs on opposite sides, making it easier to construct the constraints. A top view of the process is depicted in Figure 3. 

#### 2.2.3. Multi-Step Homogenization Procedure

**Figure 4:**
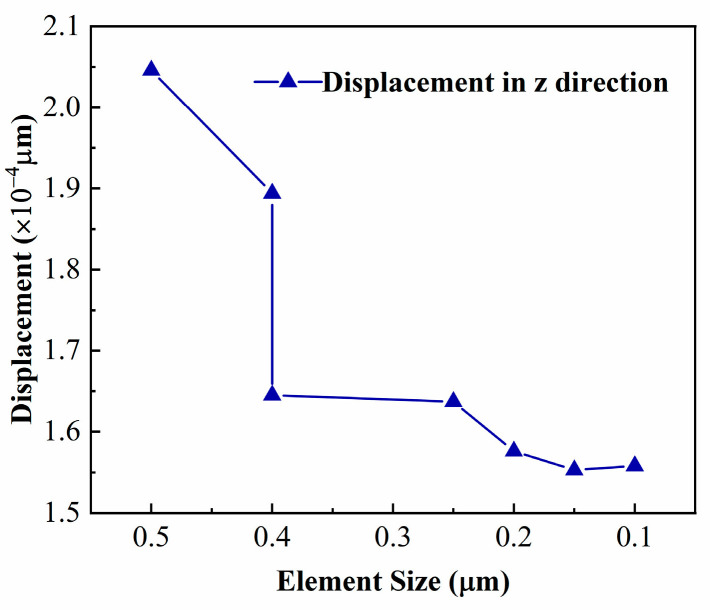
Mesh independence test by refining the mesh for the 1/4 RVE model.

The meso-scale homogenization was preceded by a mesh convergence study, a crucial step to ensure the simulation’s accuracy and efficiency. The convergence study was conducted on a 1/4 RVE model subjected to one-dimensional tensile stress. The displacement result in the z-direction was examined to verify mesh convergence, as shown in Figure 4. Subsequently, the homogenized properties were assessed, which revealed a relative error of less than 1% when the mesh converged. The mesh size determined in this step is employed in subsequent simulations. 

Since the property of the RVE in the y-direction (height) remains the same, it is concluded that the height of the RVE has no impact on the homogenized property. As a result, a smaller height was chosen for the RVE to minimize computational costs.

**Figure 5:**
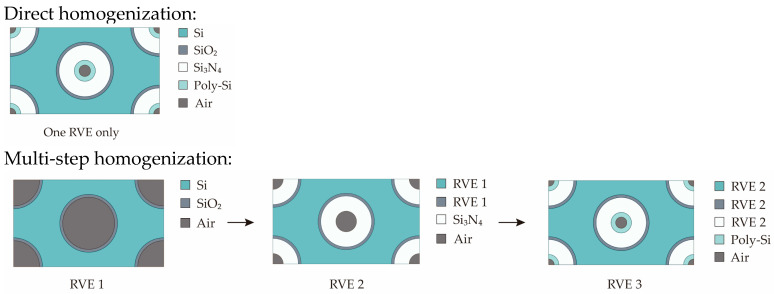
Schematic of two homogenization methods.

Regarding the detailed homogenization process, since different layers are deposited at different temperatures, the numerical homogenization is conducted using two different methods: direct homogenization and multi-step homogenization. For ease of comparison, in Section 3.2 they are also referred to as homo 1 and homo 2, respectively. This paper focuses on investigating the wafer warpage values after the deposition of SiO_2_ (process step 1) and all layers (process step 3). The homogenization methods used to determine the homogenized properties after step 1 are identical for both methods. However, the difference lies in the assignment of material properties after steps 2 and 3: The homogenization process of the two methods is depicted in Figure 5.

- In direct homogenization (homo 1), the RVE is homogenized by using the properties of each material as listed in Table 1;
- In multi-step homogenization (homo 2), after process step 2, the RVE 2 consists of three materials: Si, SiO_2_, and Si_3_N_4_. At this stage, the properties of SiO_2_ and Si are substituted with the homogenized RVE 1 determined in the previous step. Then, after process step 3, the material properties of Si, SiO_2_, and Si_3_N_4_ are substituted with the homogenized properties of the RVE 2 as determined in the last step.

The deposition process has a direct impact on subsequent steps, affecting the warpage of the wafer. While direct homogenization is commonly used for deriving properties, it overlooks the influence of previous deposition steps. Multi-step homogenization addresses this by including the impact of previous steps in subsequent ones, resulting in a more comprehensive determination of properties.

#### 2.2.4. Numerical Prediction of Wafer Warpage at the Macro-Scale 

Based on the homogenized properties obtained at the meso-scale, we performed the wafer warpage simulation at the macro-scale. In this scale, we distinguished between two layers. The upper layer is referred to as the via layer. The homogenized properties were applied to this via layer. The lower layer is designated as the substrate layer, comprising the silicon substrate.

The wafer exhibited geometric symmetry, enabling us to create a quarter-sized model and apply symmetry boundary conditions in the x and z directions, which significantly reduced the computational resources required. Additionally, to avoid rigid body motion, the central edge of the wafer was fixed.

We examined and compared the wafer warpage results at the first and final steps with experimental data. The initial wafer warpage was simulated as a temperature drop from the SiO_2_ layer’s deposition temperature of 1100 °C to room temperature. The final wafer warpage was simulated from a simplified equivalent stress-free temperature to room temperature. Using the final step temperature to simulate the cooling process has proven effective compared to adopting the whole cycle [34,35]. It is important to note that in this case, the final step temperature was different from the poly-Si deposition temperature. Given the intricate nature of the physical and chemical processes, determining the stress-free temperature at the final step necessitated a trial and error approach, as elaborated in [16]. In this trial and error process, we explored six different stress-free temperatures, ultimately selecting the temperature that closely matched the experimental results. Consequently, we selected 800 °C as the final step temperature for wafer warpage simulation.

## 3. Results and Discussions

### 3.1. Numerical Validation

**Figure 6:**
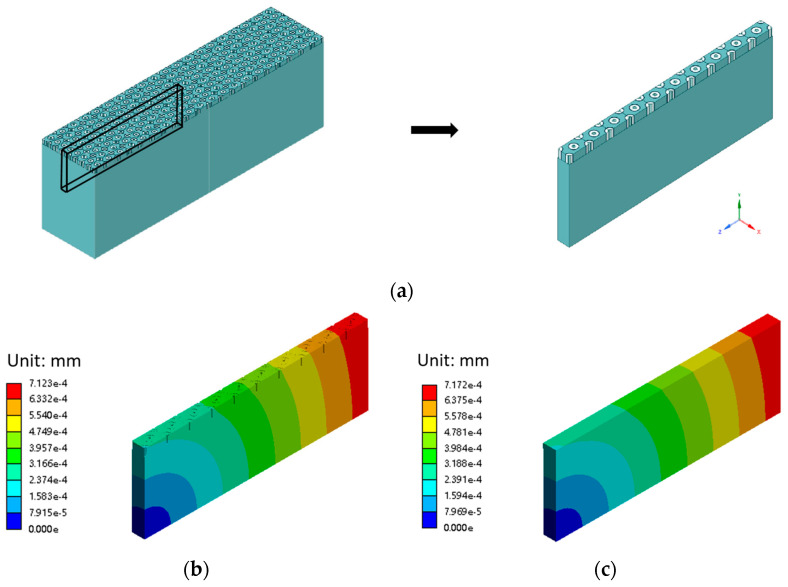
(**a**) Detailed model in the numerical validation; (**b**) Deformation of the detailed model; (**c**) Deformation of the homogenized model.

To validate the accuracy of the proposed approach, we created a numerical validation structure that had ten vias, as depicted in Figure 6a. The dimensions of the vias matched the parameters elaborated in Section 2.1, except for a via depth of 5 μm and a substrate thickness of 50 μm. The detailed model incorporated the materials listed in Table 1, while the homogenized model simplified the structure to include only Si and one homogenized material determined by the multi-step homogenization method. Identical meshing and boundary conditions were applied to the detailed and homogenized models. Both models were subjected to the same temperature variation of 1 °C. The simulation results of the homogenized model and the detailed model with the actual vias were compared. As illustrated in Figure 6b,c, the results showed insignificant differences between the deformation data of the two models, falling within a range of less than 1%. These minor differences supported the effectiveness of our method.

### 3.2. Experimental Validation

Wafer warpages were measured using the FST 5000 Film Stress Tester (SuPro Instruments, Shenzhen, China). These measurements were taken after the deposition of Si and poly-Si, respectively. Since the outer 20% of the wafer typically contains noise and is considered less significant than the inner 80%, the warpage values were tested and evaluated within the range of 15 mm to 135 mm of the wafer. Specifically, the warpage values at the 15 mm and 135 mm positions were calibrated as 0. 

**Figure 7:**
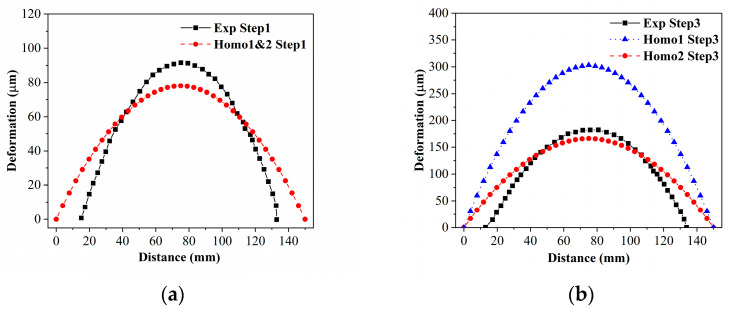
Experimental measurement of wafer warpage compared with two homogenization results (Exp: experiment results, homo 1: direct homogenization results, homo 2: multi-step homogenization results). (**a**) After process step 1: SiO_2_ layer deposition; (**b**) After process step 3: Poly-Si layer deposition.

Homogenized properties were obtained by applying the aforementioned homogenization methods and boundary conditions. The homogenized CTEs and simulated wafer warpages are displayed in Table 2 and Figure 7, respectively. Notably, the homogenized CTEs following process step 1 are identical because of the identical RVEs of the two homogenization methods after step 1.

Due to the lower homogenized CTE in the upper layer compared to the bottom layer, the warpage shape is concave, as observed in both experiment and simulation results. The numerical results are extracted from the central line of the wafer, spanning from 0 mm to 150 mm. Regarding the peak warpage value after step 1, the difference between the homogenization and experiment results is within 5%. Similarly, the difference between the multi-step homogenization and experiment results after step 3 is also within 5%. However, it should be noted that the error between the direct homogenization and experiment results after step 3 is relatively larger.

In conclusion, our homogenization method enables the numerical prediction of wafer warpage values without relying solely on experiments. This approach can help semiconductor companies save on experimental costs and provide valuable design guidance for wafer design.

### 3.3. Theoretical Analyses of the CTE Mismatch 

In layered systems, a crucial concept to consider is misfit strain, which represents the disparity in stress-free dimensions between two or more bonded layers. Various factors contribute to the generation of misfit strain, encompassing phase transformation, plastic deformation, and creep. In the context of capacitors, differential thermal contraction is one of the most influential factors. This phenomenon arises due to the difference in the CTE between the layers. During the cooling process, one layer will contract more than the other, thus causing internal stresses and strains. Since there is no externally applied force within the system, the forces acting on the two layers must balance to achieve equilibrium. This equilibrium entails tensile stress in one layer and compressive stress in another. Moreover, moment balance must also be maintained simultaneously, as the stresses in the layers induce a bending moment that tends to create curvature in the plane. 

**Figure 8:**
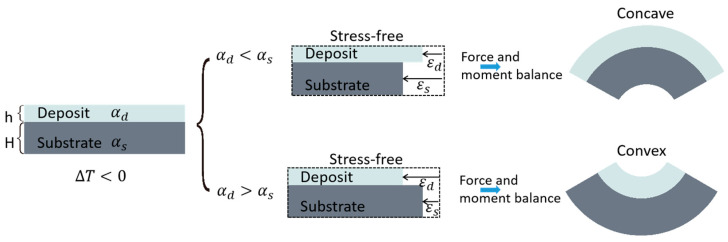
Relationship between CTE and bow shape.

An equal biaxial stress state is generated when the material used in the layered systems has isotropic properties within the plane and negligible through-thickness stress. This state can be described by introducing strain in two arbitrary in-plane directions that are orthogonal and equivalent. To provide a simplified illustration of the relationship between the CTE difference and the warpage shape, we will focus on the one-dimensional case, which could easily be generalized to higher-dimensional cases. The CTE of the deposition layer is denoted as $\alpha_{d}$ and that of the substrate is $\alpha_{s}$. When $\alpha_{d}<\alpha_{s}$, the deposition layer will contract less during cooling (see Equation (11)), resulting in a concave wafer warpage. Conversely, when $\alpha_{d}>\alpha_{s}$, the deposition layer will contract more than the substrate, leading to the formation of a convex wafer warpage, as depicted in Figure 8.

### 3.4. Parameter Sensitivity Analyses and Optimization 

**Figure 9:**
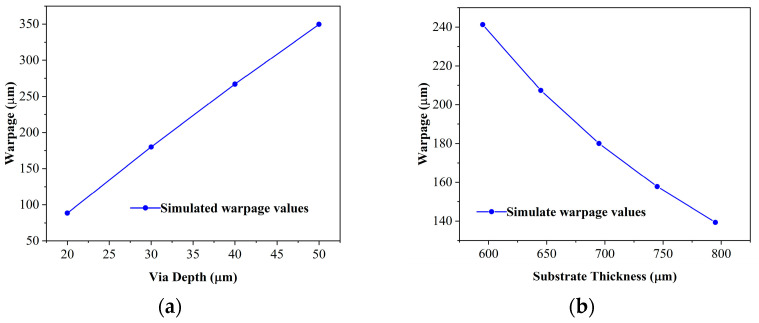
The effect of (**a**) via depth, (**b**) substrate thickness on wafer warpage.

With the presented homogenization method successfully validated by numerical and experimental results, this method was then employed to investigate the impact of substrate thickness and via depth on wafer warpage while maintaining other fixed parameters. It was observed that as via depth increased, the wafer warpage also increased (Figure 9a). Conversely, as substrate thickness increased, the wafer warpage decreased (Figure 9b). Deeper via resulted in a higher capacitance density, but it also led to an increased warpage value. Therefore, via depth should strike a balance between warpage and capacitance density. On the other hand, a thicker substrate can decrease the wafer warpage, but it also results in a thicker substrate, which is not desirable in the industry application. Therefore, there are limitations to how much the substrate thickness can be increased.

**Figure 10:**
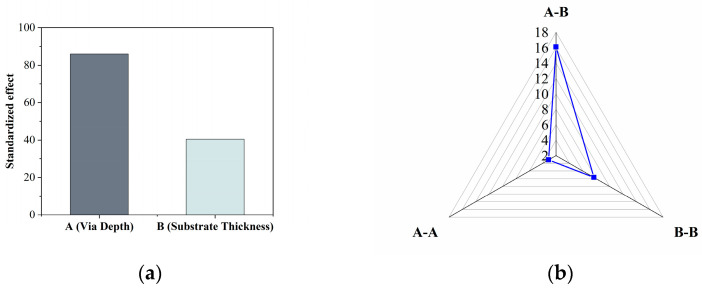
Standardized effects of (**a**) A: via depth and B: substrate thickness, (**b**) coupling factors A–A, A–B, and B–B.

It can be observed from Figure 10a that both the via depth and the substrate thickness have a noticeable impact on the wafer warpage, with the via depth exhibiting a more significant effect. When considering second-order effects and interaction terms, as illustrated in Figure 10b, the influence of the interaction term A–B is more pronounced than that of the second-order effects. This suggests that when the via depth and substrate thickness change simultaneously, they collectively impact the wafer warpage substantially.

## 4. Conclusions

This study devises and validates a multi-step homogenization method for predicting wafer warpage in silicon substrates with vias. The numerical process involves substituting the intricate physical process with a simplified temperature drop from the equivalent stress-free temperature and utilizing a homogenization method to replace the via layer with a homogenized material. Notably, the presented multi-step homogenization method differs from the conventional approach by incorporating prior RVE results into the subsequent steps. This novel method considers the influence of the previous step, thereby delivering more reliable results. The validity of both simplifications has been confirmed through numerical modeling and experimental measurements.

Furthermore, parameter sensitivity analyses were conducted to investigate the influence of various factors. It has been observed that increasing the via depth can enhance capacitance density, but it also results in a more considerable wafer warpage. Therefore, via depth should strike a balance between warpage and capacitance density. Both the via depth and substrate thickness have an impact on the wafer warpage, with the via depth being the more influential factor. Overall, applying the presented homogenization method has enabled us to estimate wafer warpages reliably and efficiently.

## Figures and Tables

**Table 1:** Materials used in the simulation.

Material	Young’s Modulus (GPa)	Poisson’s Ratio	CTE (ppm/K)
SiO_2_	69 [22]	0.14 [23]	0.5 [24]
Si_3_N_4_	290 [25]	0.27 [26]	3.4 [27]
Polysilicon	169 [28]	0.22 [28]	2.8 [29]
Silicon	161 [30]	0.28 [30]	4.4 [31]

**Table 2:** Homogenized CTEs used in the simulation.

Method	CTE (ppm/K) after Process Step 1	CTE (ppm/K) after Process Step 3
Direct homogenization	$\alpha_{x}=4.21$ $,\;\alpha_{y}=4.23$ $,\;\alpha_{z}=4.22$	$\alpha_{x}=3.86,\;\alpha_{y}=3.84,\;\alpha_{z}=3.86$
Multi-step homogenization	$\alpha_{x}=4.21$ $,\;\alpha_{y}=4.23$ $,\;\alpha_{z}=4.22$	$\alpha_{x}=3.88,\;\alpha_{y}=3.74,\;\alpha_{z}=3.88$

## Data Availability

Data are contained within the article.
